# Favipiravir—Tautomeric and Complexation Properties in Solution

**DOI:** 10.3390/ph16010045

**Published:** 2022-12-28

**Authors:** Vera Deneva, Sofia Slavova, Alina Kumanova, Nikolay Vassilev, Daniela Nedeltcheva-Antonova, Luidmil Antonov

**Affiliations:** 1 Institute of Electronics, Bulgarian Academy of Sciences, 1784 Sofia, Bulgaria; 2 Institute of Organic Chemistry with Centre of Phytochemistry, Bulgarian Academy of Sciences, 1113 Sofia, Bulgaria; 3 Institute of General and Inorganic Chemistry, Bulgarian Academy of Sciences, 1113 Sofia, Bulgaria

**Keywords:** favipiravir, COVID-19, complexation, DFT, UV–Vis, NMR

## Abstract

The tautomeric properties of favipiravir were investigated experimentally for the first time by using molecular spectroscopy (UV–Vis absorption, fluorescence and NMR), as well as DFT quantum–chemical calculations. According to the obtained results, the enol tautomer is substantially more stable in most of the organic solvents. In the presence of water, a keto form appears to be favored due to the specific solute–solvent interactions. Upon the addition of alkaline-earth-metal ions, deprotonation and complexation occurred simultaneously, giving the formation of 2 : 1 ligand : metal complexes. According to the theoretical simulations, the metal ion is captured between the carbonyl groups as a result of the size–fit effect.

## 1. Introduction

The tautomerism is a dynamic equilibrium between two or more structurally different forms with different properties of the compound in which the proton transfer occurs. This phenomenon has been considered to be very important in the drug design [1,2,3,4,5], since a large part of the active substances used in pharmacy tend to tautomerize, thus putting their biological activity and effectiveness in connection with their tautomeric state. For this reason, the possibilities for evaluating the position of the tautomeric equilibrium, along with the properties and the structure of the single tautomers, have been actively studied [6,7,8,9,10,11,12]. However, up until now, there have been no detailed experimental data about the tautomeric behavior of its various thermodynamic and kinetic effects in solution. Very precise human intervention is required to discover the appropriate conditions under which a shift of the equilibrium to the more efficient tautomeric form could be achieved.

The appearance of the SARS-CoV-2 virus was crucial for our global society, causing a widespread pandemic with devastating health, social and economic challenges for the communities worldwide. This has posed a need for vital scientific breakthroughs, which include innovative approaches to developing vaccines and antiviral drugs. The Japanese anti-influenza drug favipiravir [13,14,15,16,17,18] has shown promising results in the treatment of the disease in China, India, Russia and other countries [19,20,21,22,23,24,25], either alone [26,27,28,29,30] or in combination [31,32,33,34,35,36] with other antiviral compounds.

Favipiravir (**1**) belongs, from one side, to the 2-hydroxy pyridine family, and that is the reason why it is expected to exhibit ground-state tautomerism [37,38,39,40,41,42,43]. On the other side, it contains the structural motif of the salicylamide, which brings excited-state proton-transfer properties [44,45,46,47,48]. Up to now, the tautomeric properties of **1** and its modifications, mainly in the ground state, have been investigated theoretically under implicit solvation or in vacuum, showing a variety of theory-dependent results [49,50,51,52,53,54,55]. Therefore, the aim of this study was to conduct, for the first time, detailed experimental research concerning the tautomerism of **1** in solution by using molecular spectroscopy (UV–Vis absorption, fluorescence and NMR), as well as quantum–chemical calculations. The main emphasis is given to the effect of the solvents and the addition of metal ions in solution.

## 2. Results and Discussion

### 2.1. Tautomerism of Favipiravir

**Figure 1:**
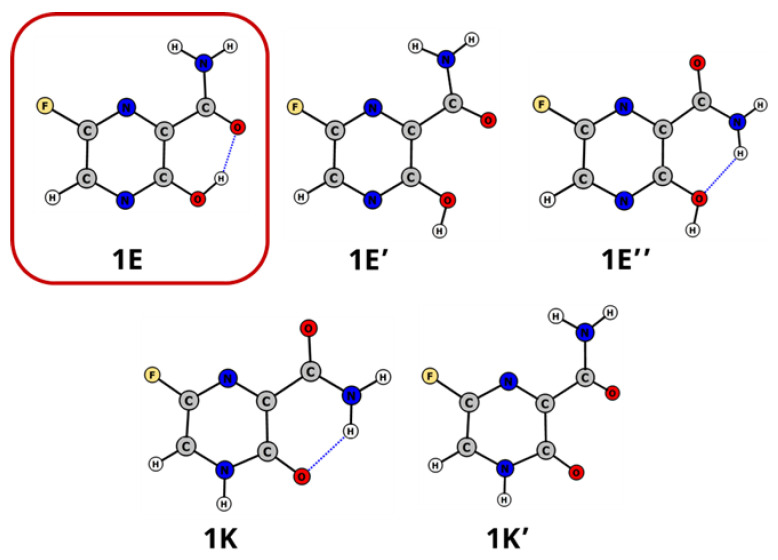
The most stable structures (M06-2X/def2-TZVPPD) of **1** in acetonitrile in the ground state (S_0_)—**E** corresponds to the enol forms, while **K** corresponds to the keto tautomers.

Favipiravir is a very complex polyfunctional tautomeric system. It contains two potentially labile protons and several proton acceptor/donor sites. The rotation of the amide and hydroxyl groups provides additional options for each of the tautomers to exist as several isomers. All logically possible structures were screened at the M06-2X/def2-TZVP level of theory (see Supplementary Table S1) and, as seen, proton exchange related to the amide nitrogen atom is unlikely to occur. According to the expectations, a ground-state tautomerism involving the two most probable tautomers with their isomers (Figure 1) should be considered only. The structures, as shown in Figure 1, were optimized by using M06-2X/def2-TZVPPD level of theory. Although, the proton transfer between the conjugated N and O atoms (from **1E** to **1K**, Figure 1) corresponds, in general terms, to iminol–amide tautomerism [56], in the discussion below, for the sake of simplicity, enol and keto are used to note **1E** and **1K**.

The corresponding relative energies of the structures (shown in Figure 1) are collected in Supplementary Table S2. In the case of the enol forms, the position of the OH proton is of importance, while the stabilization of the keto form depends on the orientation of the amide group. As a result, the sufficient stabilization in the case of hydrogen bonding formation makes the **1E** and **1K** isomers, featuring intramolecular hydrogen bonding, most stable among the other possible isomers (see Supplementary Tables S1 and S2).

Considering the data shown in Table 1, it can be clearly seen that the enol tautomer is significantly more stable than the keto one, thus suggests that, in solution, favipiravir exists predominantly in this form. As for the keto form, the very high relative energy values are an indication that it would hardly exist in solution, although the increase of the solvent polarity leads to some stabilization due to its larger dipole moment.

The predicted spectral data suggest that the shift from the enol to the keto tautomer should be accompanied by a red shift in the absorption spectra and up field of the tautomeric proton in ^1^H NMR. The absorption spectra of favipiravir in toluene, chloroform and acetonitrile are shown in Supplementary Figure S1. As seen, the change of the solvent from low to high polarity does not lead to significant spectra changes (both as position and as shape), thus confirming the conclusion made above, about the presence of the enol form only in solution. The ^1^H NMR spectra in acetonitrile also show only an OH signal at 12.82 ppm, as is typical for a proton involved in intramolecular hydrogen bonding.

**Figure 2:**
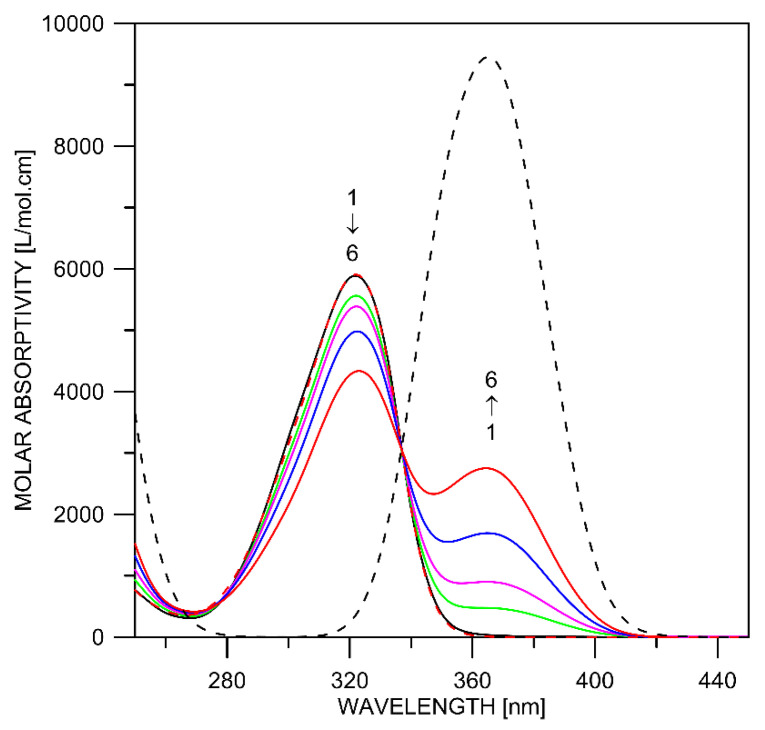
Absorption spectra of favipiravir in acetonitrile/water mixtures with stepwise decrease of the acetonitrile content: (1) 100% acetonitrile (0% water, black solid line), (2) 80% acetonitrile (20% water, green solid line), (3) 60% acetonitrile (40% water, magenta solid line), (4) 40% acetonitrile (60% water, blue solid line), (5) 20% acetonitrile (80% water, red solid line) and (6) calculated spectrum of the keto form (black dash line). The calculated spectrum of the pure enol form (red dash line) fully overlaps with curve (1).

As seen from Figure 2, the addition of water to the acetonitrile solution leads to a change in the absorption spectra—the intensity of the band at 320 nm decreases with increased water content. At the same time, a new broadband appears at 365 nm. It is worth noting that this process is concentration independent and cannot be attributed to formation of associates in the solvents used. Three possible processes could be expected in this case: shift of the tautomeric equilibrium towards the keto tautomer, stabilization of **1E″** by forming complex with water molecules and deprotonation of favipiravir.

**Figure 3:**
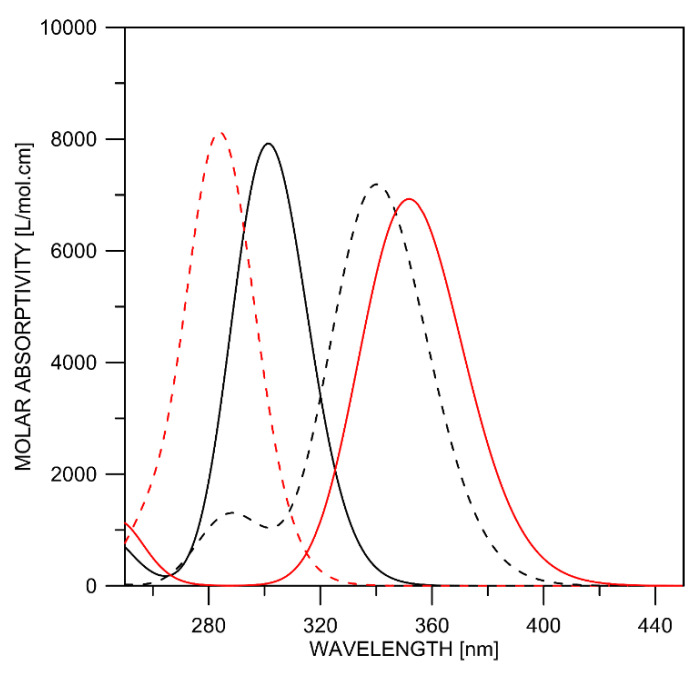
Simulated absorption spectra (B3LYP/def2-TZVPPD//M06-2X/def2-TZVPPD) in acetonitrile: **1E** (black solid line), **1K** (black dashes), **1E″** (red dashes) and the most stable (Supplementary Table S3) deprotonated form (red solid line).

In order to facilitate the discussion, the predicted spectra of **1E**, **1K**, **1E″** and the most stable deprotonated form (see Supplementary Table S3) are shown in Figure 3. As seen from Figure 2 and Figure 3 and Table 1, in the case of **1E**, the simulated band position is hypsochromically shifted in respect of the experimentally measured absorption maximum. The same behavior could be expected for the spectra of the rest of the structures.

According to the theoretical simulations, the obtainment of **1E″** should be accompanied by a blue shift in the spectra, which is not observed. The existence of the OH group in the enol forms gives a possibility for partial deprotonation with the addition of water. The deprotonation in acetonitrile with the addition of triethyl amine also leads to a red shift in the absorption, with appearance of a new band at 371 nm (Supplementary Figure S2 and Table S3). However, this new band has a different shape compared with the band at 365 nm from Figure 2. The second derivative spectra (shown in Supplementary Figure S3) clearly indicate this fact—the band with maximum at 371 nm consists of two sub-bands. The fluorescent spectra of the products (shown in Supplementary Figures S1, S4 and S5) are also different. The theoretically simulated spectrum (Figure 3) of the deprotonated specie also suggests that it could absorb bathochromically in respect to the keto tautomer, as it is actually observed experimentally.

The discussion above, in a sufficient way, proves that the spectral changes shown in Figure 2 indicate the appearance of the keto tautomer. The newly observed band at 365 nm belongs to **1K**, and this is also in agreement with the theoretical predictions (Table 1 and Figure 3).

Taking into account that the spectrum of the keto form is unknown, it is impossible to estimate the content of the tautomers by means of classical spectrophotometric analysis [57]. Therefore, a specially developed method for the spectral analysis of tautomeric systems, based on overlapping bands decomposition, was applied [58]. The fitting procedure yielded the individual spectra of **1E** and **1K** (already shown in Figure 2), as well as the estimated values of the molar fractions of the tautomers (collected in Table 2). The molar fractions allowed us to calculate the tautomeric constants and the corresponding ΔG_298K_ values. It is worth mentioning that these quantitative data are the first ones for the tautomerism of favipiravir that have been published up until now.

**Figure 4:**
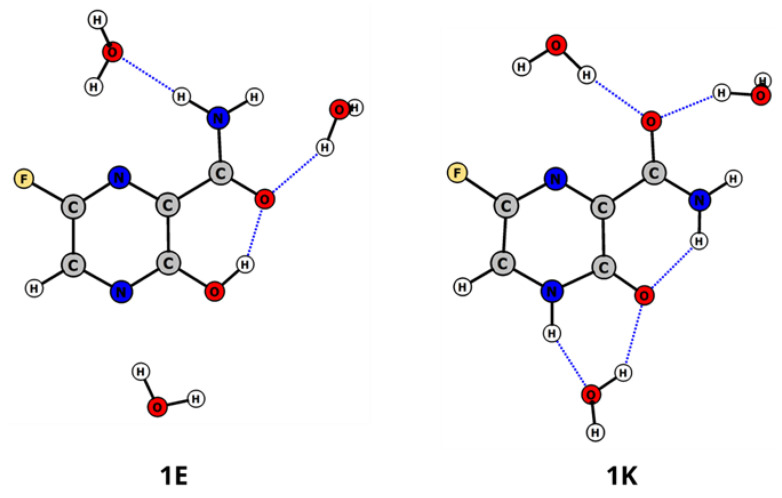
The optimized geometry (M06-2X/def2-TZVPPD) of water–favipiravir clusters with three water molecules (in acetonitrile as implicit solvent) in the ground state (S_0_) for the most stable enol **1E** and keto **1K** tautomers.

The data from Table 2 and Figure 2 show that the addition of water stabilizes the keto tautomer, in which the tautomeric proton is accessible to the solvent. A simple extrapolation based on the results from Table 2 predicts a ΔG value of −0.1 kcal/mol in pure water, suggesting that both tautomers should be presented in almost equal quantities in aqueous solution. The whole process of water–favipiravir interaction is very complex, but the tendency for the increase of water content to lead to the stabilization of the keto tautomers is obvious. According to the theoretical calculations, by using implicit solvation, the keto–enol relative energy is 6.6 kcal/mol in water, which should exclude the existence of the keto form. Obviously, there is specific stabilization due to the formation of tautomer–water complexes. As an example, the complexes of both tautomers with three water molecules are shown in Figure 4. Although the enol form remains more stable in the frame of the complex, its relative stability is reduced in respect to the keto form to 2.3 kcal/mol from 6.6 as isolated molecules (see Table 1).

On the one hand, the increase of the dielectric constant favors the stabilization of the more polar **K** tautomer (as very well seen in Table 1), and on the other hand, the availability of a free NH proton near the tautomeric C = O group and the amide carbonyl group, not involved in hydrogen bonding, leads to the formation of the associates with the water molecules and, hence, to additional stabilization. Most probably, the real situation in solution is more complex, and the sharply rising number of possible variations with increasing number of water molecules makes it impossible to follow the changes by using ordinary quantum–chemical calculations.

The emission spectra of favipiravir in the used solvents are shown in Supplementary Figures S2, S4 and S5. In toluene, acetonitrile and chloroform, where only the enol form is presented, a single weak emission at around 440 nm is observed (see Supplementary Table S4). The addition of water leads to a decrease in the emission band, but no new band originating from the **K** form appears. The Stokes shift in acetonitrile (also in toluene and chloroform) is more than 8000 cm^−1^, which suggests an excited-state intramolecular proton transfer from OH to the oxygen from the amide group. At the same time, the quantum yield is around and less than 1%, indicating the low efficiency of the phototautomeric process. The behavior is very similar to that of salicylamide, which exhibits excited-state proton transfer [44,45,46,47,48]. The theoretical calculations of **1E** in the excited state confirm this suggestion. In S_1_, a spontaneous barrierless proton transfer occurs from the Franck–Condon state of the excited enol, giving a phototautomer, the structure of which is shown in Supplementary Figure S6. Upon relaxation to the ground state, the original **1E** structure is restored through ground-state barrierless proton transfer [59]. The keto form does not possess structural changes in the excited state.

### 2.2. Complex Formation

**Figure 5:**
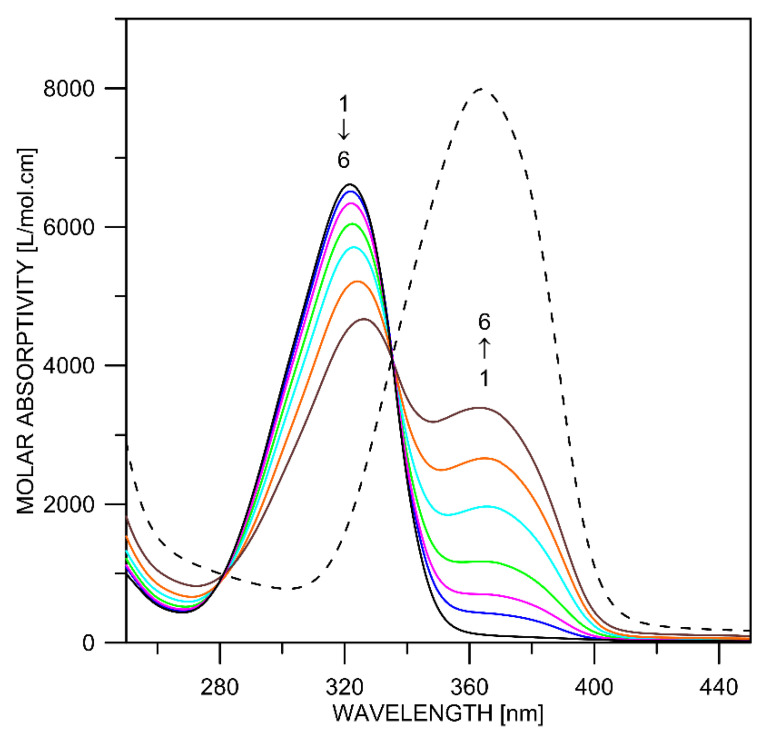
Absorption spectra of favipiravir ((c = 1.5 × 10^−5^ M)) in acetonitrile with Mg(ClO_4_)_2_ addition with molar ratios as follows: (1) (blue line) 1:0.02, (2) (magenta line) 1:0.1, (3) (green line) 1:0.2, (4) (cyan line) 1:0.5, (5) (orange line) 1:2 and (6) (brown line) 1:3. The spectra of the pure favipiravir and the calculated spectrum of the complex are shown in black solid line and in black dashes correspondingly.

The ability of **1** to form complexes in acetonitrile with alkali- and alkaline-earth-metal ions was investigated, as a model of the complexation abilities of favipiravir. In Figure 5, the spectral changes upon addition of magnesium perchlorate are shown. The appearance of a new band at ~370 nm upon the metal salt is evident. The corresponding spectra upon the addition of Li^+^, Na^+^, Ca^2+^ and Ba^2+^ as perchlorates are shown in Supplementary Figures S7–S10. As seen, the alkali metal ions do not cause any spectral changes, while Mg^2+^ and Ca^2+^ lead to the formation of complexes. No spectral changes upon the addition of Ba^2+^ were observed.

**Scheme 1:**
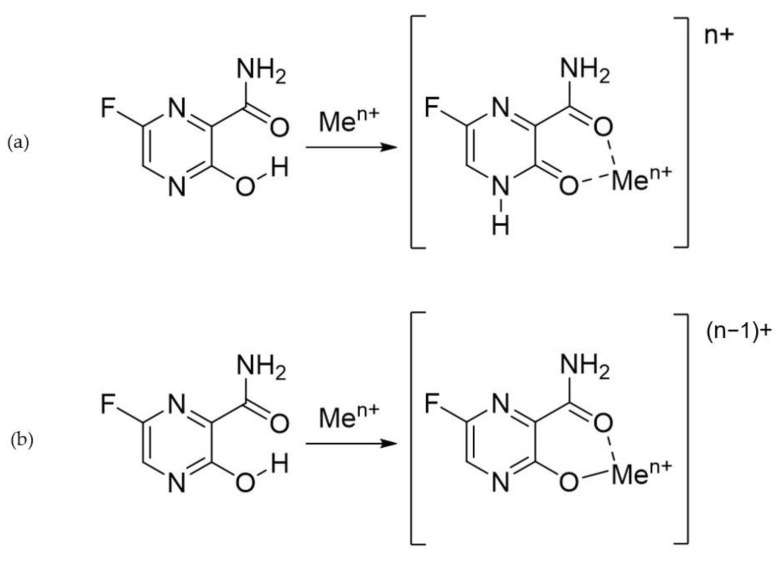
Possible scenario of **1** complexation.

Two possible hypotheses can be drawn to explain the observed complexation (Scheme 1):

(a) Simultaneous tautomeric transition and complexation: The complex is formed by interaction with the two carbonyl groups in the keto form, preceded by change in the tautomeric state from the most stable enol form.

(b) Deprotonation and complexation: The complex formation leads to deprotonation in the favipiravir molecule.

Returning back to Figure 5, the addition of magnesium perchlorate leads to the decrease of the band at around 320 nm with increased intensity at 370 nm. The spectra were processed by the chemometric procedure [58], allowing us to estimate the individual spectrum of the complex and its molar fractions (shown in Supplementary Table S5). Comparing the spectrum of the complex with the spectra of the pure keto tautomer (Figure 2) and the deprotonated favipiravir (Supplementary Figure S2), it is impossible to answer which of the abovementioned scenario is realized.

According to the data from Table 1, ^1^H NMR can distinguish between the enol and keto form, monitoring the tautomeric proton. In acetonitrile, as mentioned, only a signal at 12.82 ppm belonging to **1E** is observed in the free ligand. The addition of magnesium perchlorate leads to a decrease of this signal and increase of the water signal. The change is more remarkable in ^19^F NMR spectrum (Supplementary Figure S11), where the signal of favipiravir (−94.2 ppm, doublet with J = 7.9 Hz) is decreasing and a broad signal at −102.6 ppm appears. The substitution of enol proton with magnesium leads to a decrease in electron density on the F atom, and the ^19^F signal of complex is shifted to a higher field. This matches very well with the experimentally predicted NMR shifts—from −94.9 for the enol form to −103.0 for the deprotonated favipiravir in the complex. The pure keto tautomer is expected to have a signal at −112 ppm. These results suggest that deprotonation occurs in the process of complex formation, which can be conformed also by the obtained mass spectra.

In the ESI–HRMS spectra, acquired in positive full-scan mode in the range 100–1200 *m*/*z* range, a 337.0354 *m*/*z* ion was observed, allowing us to suggest a complex of the deprotonated favipiravir with a 2:1 ligand–metal ratio. The performed MS^2^ measurements confirmed the proposed complex structure. Therefore, from the spectra shown in Figure 5 and Figure S9, the logarithmic stability constants of the 2 : 1 ligand : metal complexes of Mg^2+^ and Ca^2+^ were estimated as 1.13 ± 0.04 and 0.75 ± 0.05, respectively.

The interaction ligand–metal ion happens in the beta dicarbonyl cavity of the ligand, making the size–fit effect very important. According to the results from the theoretical simulations (shown in Supplementary Figure S12), Li^+^, Mg^2+^ and Ca^2+^ can enter the cavity, while larger ions such as K^+^ and Na^+^ cannot. According to the stabilization energies of the complex formation (collected in Supplementary Table S6), the stabilization in the case of magnesium (II) is much larger, followed by the value of Ca^2+^, and this fits the experimental observations reasonably well.

## 3. Materials and Methods

Favipiravir was purchased from Key Organics/BioNet (Cornwall, UK), had a purity of 97% and was used without further purification. The complexation was studied in acetonitrile, dried with P_2_O_5_, distilled upon CaH_2_ and kept under molecular sieve [60]. AR-grade LiClO_4_ (Fluka), NaClO_4_.H2O (Fluka), Mg(ClO_4_)_2_ (Fluka), Ca(ClO_4_)_2_.4H_2_O (Aldrich) and Ba(ClO_4_)_2_ (Fluka) were vacuum-dried at 60 °C from 3 to 5 days, depending on the case, till constant weight.

Spectral measurements were performed on a Jasco V-570 UV–Vis–NIR spectrophotometer (Jasco Inc., Japan) that was equipped with a thermostatic cell holder (using Huber MPC-K6 thermostat (Peter Huber Kältemaschinenbau AG, Germany) with precision 1 °C) at room temperature. The steady-state fluorescence spectra were recorded with a Jasco FP-6600 spectrofluorometer (Jasco Inc., Japan) at room temperature. For spectroscopy measurements, all solvents were of spectroscopy-grade purity. The spectral changes in water and upon addition of metal perchlorates or triethyl amine were achieved by stepwise titration of isomolar solution of **1** in acetonitrile. The obtained spectra were analyzed by a quantitative procedure based on the resolution of overlapping bands, as this yields the individual spectra of the components and their molar fractions in each solution [57,58].

The stability constants (β) were estimated according to the following stoichiometric equation: 2 L+M ↔C. The mass balance kept the initial concentration of the ligand constant ($c_{0}$) and increased the concentration of the added metal salt ($c_{M}^{i}$): $c_{L}^{i}+2\;x\;\;c_{C}^{i}=c_{0}$. Stability constant as a function of the added metal salt ($c_{M}^{i}$):

$$\beta =\frac{c_{C}^{i}}{{\left(c_{L}^{i}\right)}^{2}.c_{M}^{i}}$$

where ***L*** is the ligand, ***C*** is the complex, ***M*** is the corresponding metal ion and *c* is the concentration in *i*th solution.

The NMR spectra were recorded on a Bruker Avance Neo 600 MHz spectrometer (Bruker, Germany) that was equipped with a 5 mm Prodigy probe.

Liquid chromatography–mass spectrometry analysis was carried out on a Q Exactive^®^ hybrid quadrupole-Orbitrap^®^ mass spectrometer (ThermoScientific Co., USA) that was equipped with a HESI^®^ (heated electrospray ionisation) module, TurboFlow^®^ Ultrahigh-Performance Liquid Chromatography (UHPLC) system (ThermoScientific Co., USA) and HTC PAL^®^ autosampler (CTC Analytics, Switzerland). The chromatographic separation of the analyzed compounds was achieved on an XBridge BAH Shild RP 18 (100 × 2.1 mm, 2.5 µm) analytical column (Waters, USA), using gradient elution at 300 µL/min flow rate. The used eluents were A—10 mM ammonium hydrogen carbonate; and B—buffer A/CAN (1/9, *v*/*v*). Full-scan mass spectra over the *m*/*z* range 100–1200 were acquired in positive ion mode at resolution settings of 70,000. Parallel reaction monitoring (PRM) mode at resolution settings of 17,500 and 0.5 amu isolation window of precursor ions was used for quantitative analysis. The mass spectrometer operating parameters used in a negative ionization mode were spray voltage—4.0 kV; capillary temperature—320 °C; probe heater temperature—300 °C; sheath gas flow rate—35 units; auxiliary gas flow—12 units; sweep gas—2 units (units refer to arbitrary values set by the Q Exactive Tune software); and S-Lens RF level of 50.00. Nitrogen was used for sample nebulization and collision gas in the HCD cell. Normalized collision energy was also optimized for each derivative and was in the range between 20% and 35%. All derivatives were quantified by using 5 ppm mass tolerance filters to their theoretical calculated *m*/*z* values. Data acquisition and processing were carried out with XCalibur^®^ version 2.4 software package (ThermoScientific Co., Waltham, MA, USA).

Based on the previous theoretical results at the M06-2X/def2-TZVP level of theory [49], geometry optimization of the most stable tautomeric forms of **1** in the ground state was performed by using the same DFT method with def2-TZVPPD basis set. The M06-2X [61] refers to the range-separated hybrid density functional within the generalized gradient approximation. In our previous studies of tautomeric systems [62], it was shown that the abovementioned selected functional correctly describes the relative stability of the tautomeric forms and demonstrates a good agreement between the experimental data and theoretical results. The selected basis set def2-TZVPPD [63] includes one contracted basis function to describe inner shells and three basic functions for valence shells, as well as a set of polarization functions and one diffuse function, making it an appropriate approximation tool for quantum chemical calculations of such compounds.

Bearing in mind that M06-2X systematically underestimates the absorption-band positions [64], the UV–Vis spectral data were predicted by the B3LYP [65] functional, using the M06-2X optimized ground-state geometries. The NMR chemical shieldings of selected tautomeric forms of the studied compounds were calculated by using the GIAO approximation [66]. The calculated absolute shieldings were transformed to chemical shifts, using the corresponding reference compounds.

The TD-DFT method was used for singlet excited-state optimizations, employing long-range corrected CAM-B3LYP functional [67] with def2-TZVPPD basis set.

All calculations were performed by taking into account solvation effects via polarizable continuum model (PCM formalism) [68], considering the solvent as a dielectric medium. All structures were optimized without restrictions, using tight optimization criteria and an ultrafine grid in the computation of two-electron integrals and their derivatives. The true minima were verified by performing frequency calculations in the corresponding environment. The quantum chemical calculations were performed by using the Gaussian 16 Rev. C.01. software package [69].

## 4. Conclusions

In this study, we demonstrated that the keto–enol tautomerism of favipiravir depends not so much on the dielectric constant of the solvent as on the ability of the particular tautomer to stabilize through specific interactions with the solvent. In the used organic solvents, the enol tautomer is only presented in solution, stabilized by intramolecular hydrogen bonding. The addition of water shifts the equilibrium partially to the keto tautomer, due to favored solute–solvent interactions. The absorption spectrum of the pure keto form has been observed for the first time. Upon addition of alkaline-earth-metal ions, deprotonation and complexation occur simultaneously, resulting in the formation of 2:1 ligand:metal complexes in the case of Mg^2+^ and Ca^2+^. The results indicate the importance of the size–fit effect.

## Figures and Tables

**Table 1:** Theoretically predicted (M06-2X/def2-TZVPPD) relative stability and spectral characteristics of the most stable tautomeric forms of favipiravir.

Tautomer	Solvent	ΔE(298 K) [kcal/mol]	ΔE(0 K) [kcal/mol]	ΔG(298 K) [kcal/mol]	λ_max_ *^,^** [nm]	*f* *	^1^H NMR NH/OH [ppm]
**1E**	Toluene	0.00	0.00	0.00	305	0.21	13.28
	Acetonitrile	0.00	0.00	0.00	301	0.20	13.61
	Water	0.00	0.00	0.00	301	0.20	13.63
**1K**	Toluene	10.70	10.84	10.45	340	0.18	9.09
	Acetonitrile	6.87	7.13	6.81	340	0.19	9.70
	Water	6.66	6.93	6.61	340	0.19	9.73

* The absorption spectra (position of the long-wavelength band and its oscillator strength) were predicted by using the B3LYP/def2-TZVPPD//M06-2X/def2-TZVPPD level of the theory. ** The shown values correspond to S_0_-S_2_ transition; the transition S_0_-S_1_ has an oscillator strength of zero.

**Table 2:** Parameters of the **1E⇌1K** tautomeric equilibrium as a function of the water content in acetonitrile/water binary mixture at 298 K.

Water [vol. %]	1E [%]	1K [%]	K_T_ *	ΔG_298K_ [kcal/mol]
0	100	0	0.00	-
20	95	5	0.05	−1.74
40	90	10	0.11	−1.30
60	82	18	0.22	−0.90
80	71	29	0.41	−0.53

* Defined as [**1K**]/[**1E**] ratio.

## Data Availability

Data is contained within the article and Supplementary Material. The optimized structures, obtained by the theoretical calculations, are available as cartesian coordinates on request from the authors.

## Supplementary Materials

The following supporting information can be downloaded at https://www.mdpi.com/article/10.3390/ph16010045/s1. Figure S1: Absorption (up) and emission (down, c = 1.5 × 10^−5^ M, λ_ex_ = 320 nm) spectra of favipiravir in toluene (magenta line), acetonitrile (black line) and chloroform (green line); Figure S2: Absorption spectra of favipiravir (c = 1.5 × 10^−5^ M) in acetonitrile with addition of triethylamine with molar ratios as follows: 1 (black line)—no triethylamine addition, 2 (blue line)—1:0.002, 3 (red line)—1:0.02, 4 (purple line)—1:2, 5 (brown line)—1:3, 6 (green line)—1:5, 7 (magenta line)—1:7, 8 (orange line)—1:10, 9 (black dash line)—1:12; Figure S3: Second derivative spectra of the curves corresponding to the deprotonated **1** in acetonitrile (black line, derived from curve 9 from Figure S2) and **1** with maximum water addition (blue line, derived from curve 5 from Figure 2); Figure S4: Emission spectra of **1** in acetonitrile (black line) with excitation at 360 nm, in acetonitrile with TEA addition (black dash line) with excitation at 370 nm, in acetonitrile/water mixture: 20%/80% (blue line) with excitation at 320 nm and in acetonitrile/water mixture: 20%/80% (red line) with excitation at 360 nm; Figure S5: Excitation spectra of **1** in acetonitrile (black line) with emission at 440 nm, in acetonitrile with TEA addition (black dash line) with emission at 430 nm and in acetonitrile/water mixture: 20%/80% (blue line) with emission at 440 nm; Figure S6: The optimized structures of enol form **1E** of favipiravir in the ground state (S_0_, M06-2X/def2-TZVPPD) and its phototautomer in the excited state (S_1_, CAM-B3LYP/def2-TZVPPD). The calculations are done in acetonitrile; Figure S7: Absorption spectra of favipiravir (c = 1.5 × 10^−5^ M) in acetonitrile with LiClO_4_ addition with molar ratios as follows: 1 (black line)—100% acetonitrile, 2 (blue line)—1:2, 3 (red line)—1:3; Figure S8: Absorption spectra of favipiravir (c = 1.5 × 10^−5^ M) in acetonitrile with NaClO_4_ addition with molar ratios as follows: 1 (black line)—100% acetonitrile, 2 (blue line)—1:2, 3 (red line)—1:3; Figure S9: Absorption spectra of favipiravir (c = 1.5 × 10^−5^ M) in acetonitrile with Ca(ClO_4_)_2_ addition with molar ratios as follows: 1 (black line)—100% acetonitrile, 2 (orange line)—1:0.002, 3 (blue line)—1:0.02, 4 (red line)—1:0.1, 5 (green line)—1:0.2, 6 (cyan line)—1:0.5, 7 (purple line)—1:1; Figure S10: Absorption spectra of favipiravir (c = 1.5 × 10^−5^ M) in acetonitrile with Ba(ClO_4_)_2_ addition with molar ratios as follows: 1 (black line)—100% acetonitrile, 2 (red line)—1:0.002, 3 (blue line)—1:0.02, 4 (magenta line)—1:2, 5 (green line)—1:3; Figure S11: 19F NMR spectra of favipiravir in acetonitrile-d3 with increasing addition of Mg(ClO_4_)_2_ from bottom to top; Figure S12: Theoretically predicted (M06-2X/def2-TZVPPD) most suitable sites for favipiravir complexation with Li^+^, Na^+^, K^+^, Mg^2+^ and Ca^2+^ in acetonitrile in the ground state (S0); Table S1: Relative energies of the possible tautomers and their isomers in acetonitrile (M06-2X/def2-TZVP); Table S2: Relative energies of the isomeric forms of the enol and keto tautomers obtained by using M06-2X/def2-TZVPPD level of theory (see Figure 1 in the main text); Table S3: Relative energies (M06-2X/def2-TZVPPD) of the deprotonated forms of favipiravir in acetonitrile; Table S4: Emission maxima and quantum yields of favipiravir in different solvents; Table S5: Molar fractions of the Mg^2+^ complex with **1** in acetonitrile (Figure 5); Table S6: Stabilization energy (M06-2X/def2-TZVPPD, in acetonitrile) of the metal complexes with favipiravir, compared with the ion radiuses of the metal ions (the energy is calculated as *E_stab_ = (E_lig_ + E_Me_) − E_complex_*)).
