# Supplementary material for: Favipiravir—Tautomeric and Complexation Properties in Solution
Source: Pharmaceuticals (Basel). 2022 Dec 28;16(1):45. doi: 10.3390/ph16010045 (PMC9864296; doi:10.3390/ph16010045)
Supplement: Supplementary file 1 [file pharmaceuticals-16-00045-s001.zip › pharmaceuticals-2049512-supplementary.pdf]

Supporting Information

Favipiravir – tautomeric and complexation properties in solution

Vera Deneva[1,2,*], Sofia Slavova[1,3], Alina Kumanova[1], Nikolay Vassilev[2], Daniela Nedeltcheva[1,2] and Luidmil Antonov[1,*]

[1] Institute of Electronics, Bulgarian Academy of Sciences, 1784 Sofia, Bulgaria

[2] Institute of Organic Chemistry with Centre of Phytochemistry, Bulgarian Academy of Sciences, 1113 Sofia, Bulgaria

[3] Institute of General and Inorganic Chemistry, Bulgarian Academy of Sciences, 1113 Sofia, Bulgaria

* Correspondence authors

Table S1. Relative energies of the possible tautomers and their isomers in acetonitrile (M06-2X/def2-TZVP).

Structure	ΔE(298K) [kcal/mol]	ΔE(0K) [kcal/mol]	ΔG(298K) [kcal/mol]
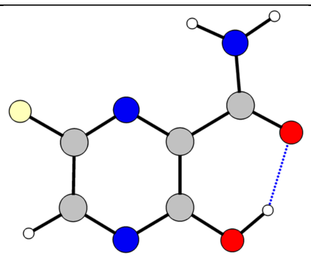	0.00	0.00	0.00
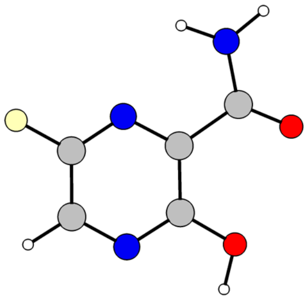	6.67	6.47	5.79
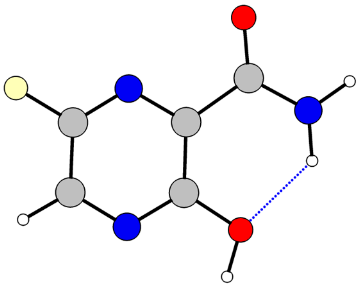	7.17	6.97	6.07
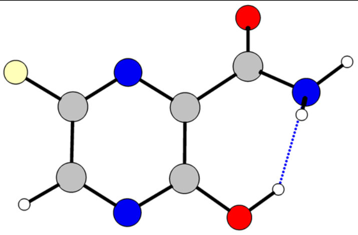	10.71	10.84	10.60

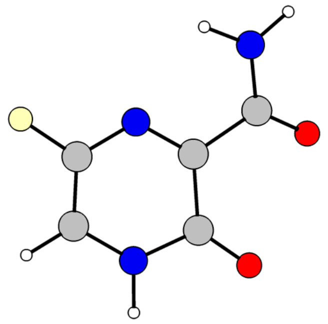	9.31	9.35	8.63
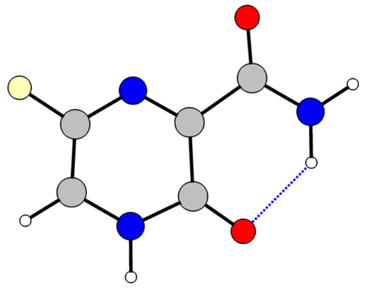	6.53	6.82	6.49
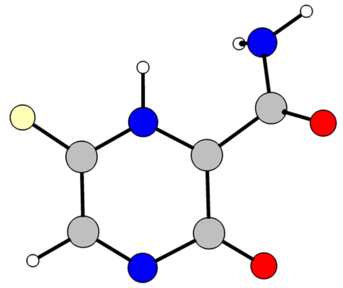	31.59	31.59	31.06
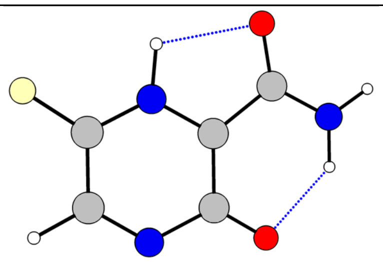	18.83	19.02	18.96
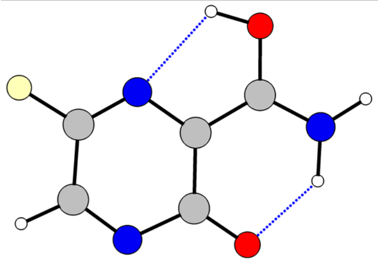	13.38	13.45	13.57

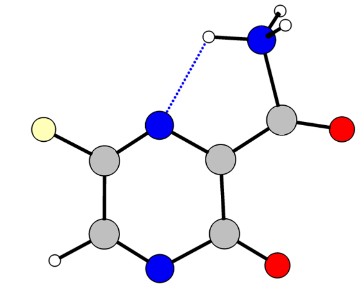	30.94	30.75	30.51
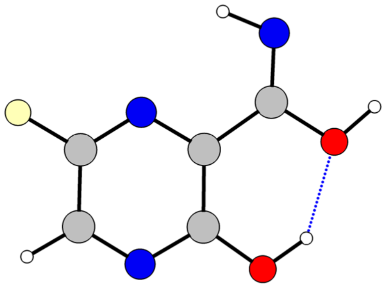	19.00	19.07	18.84
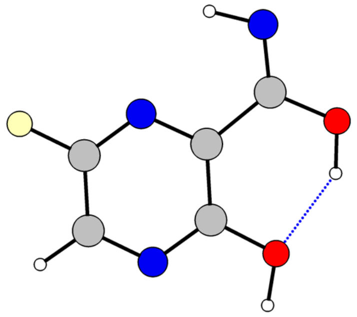	21.25	21.15	20.64
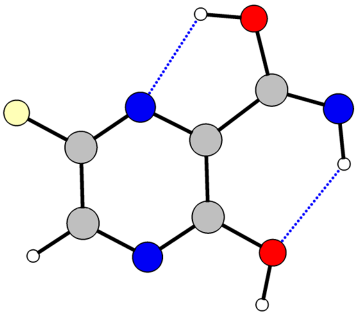	19.92	20.13	19.94
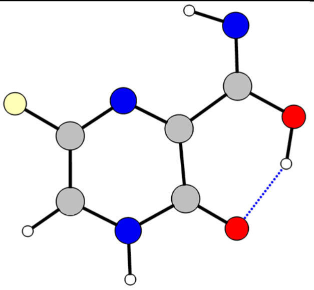	19.11	19.54	19.46

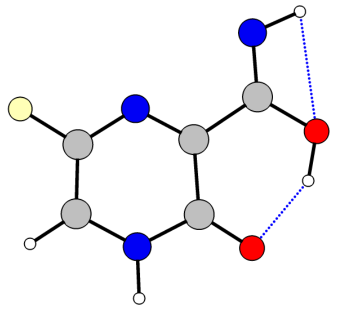	20.37	20.58	20.46
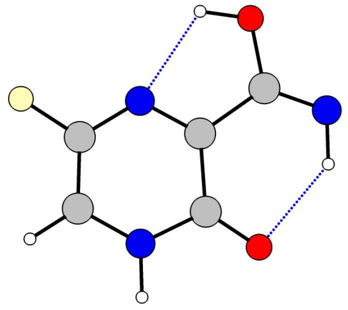	19.90	20.50	20.40
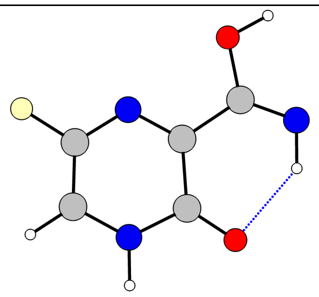	20.92	21.36	21.06
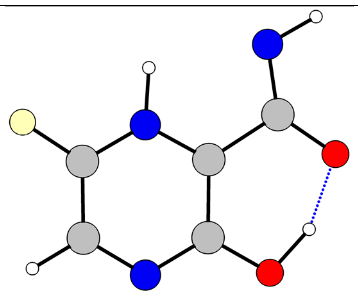	34.18	33.32	33.50
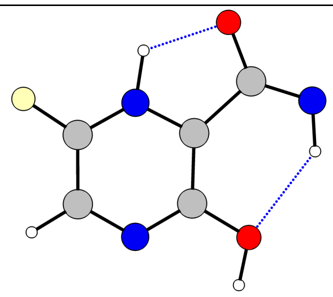	44.44	43.79	43.56

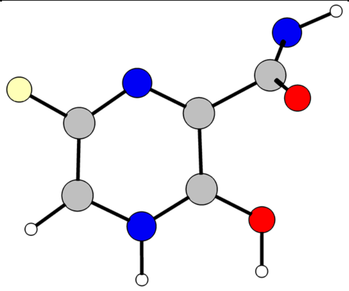	52.05	51.95	51.33

Table S2: Relative energies of the isomeric forms of the enol and keto tautomers obtained by using M06-2X/def2-TZVPPD level of theory (see Figure 1 in the main text).

**Compound**	ΔE(298K) [kcal/mol]	ΔE(0K) [kcal/mol]	ΔG(298K) [kcal/mol]
**1E**	0.00	0.00	0.00
**1E’**	6.86	6.62	5.90
**1E’’**	7.26	7.05	6.23
**1K**	6.87	7.13	6.81
**1K’**	9.79	9.76	9.05

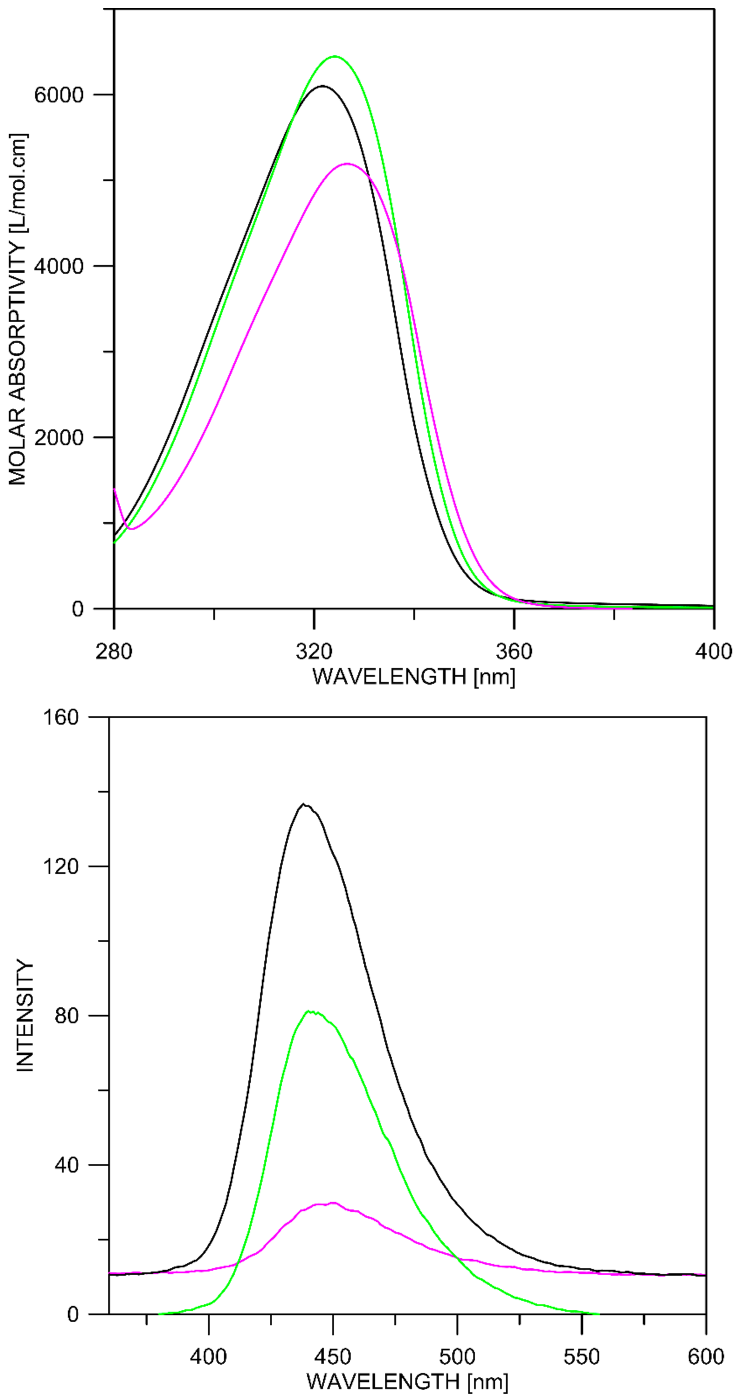

Figure S1. Absorption (up) and emission (down, c=1.5 x 10^{-5} M, λ_{ex}=320 nm) spectra of favipiravir in toluene (magenta line), acetonitrile (black line) and chloroform (green line).

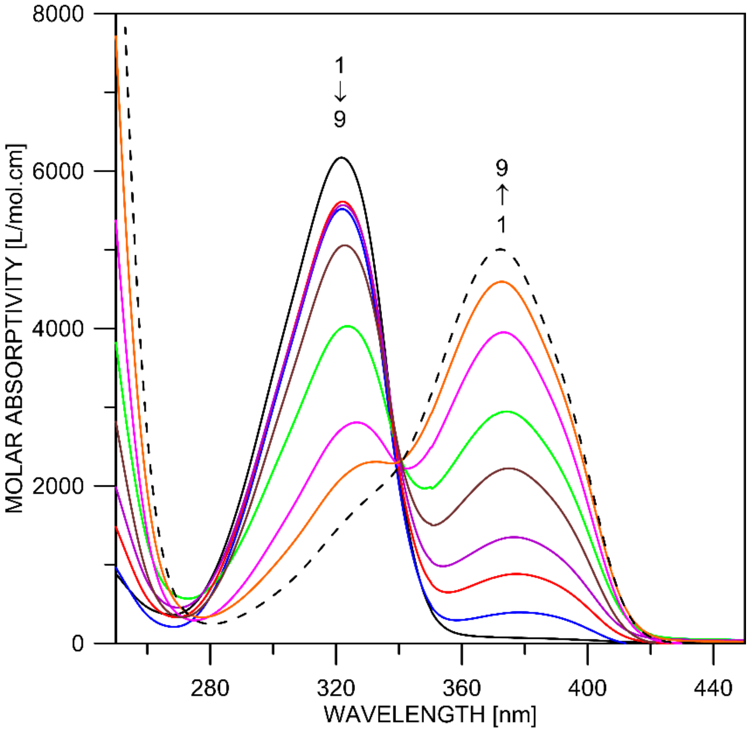

Figure S2. Absorption spectra of favipiravir (c=1.5 x 10^{-5} M) in acetonitrile with addition of triethylamine with molar ratios as follows: 1 (black line) – no triethylamine addition, 2 (blue line) – 1:0.002, 3 (red line) – 1:0.02, 4 (purple line) – 1:2, 5 (brown line) – 1:3, 6 (green line) – 1:5, 7 (magenta line) – 1:7, 8 (orange line) – 1:10, 9 (black dash line) – 1:12.

Table S3. Relative energies (M06-2X/def2-TZVPPD) of the deprotonated forms of favipiravir in acetonitrile.

**Deprtonated structure**	ΔE(298K) [kcal/mol]	ΔE(0K) [kcal/mol]	ΔG(298K) [kcal/mol]	λ_{max}* (S_0-S_1) [nm]	*f** (S_0-S_1)	λ_{max}* (S_0-S_2) [nm]	*f** (S_0-S_2)
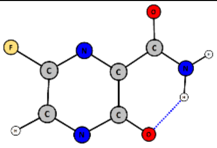	0.00	0.00	0.00	352	0.18	341	0.00
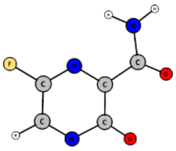	5.43	5.04	4.45				
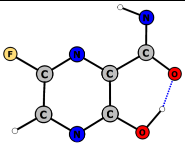	9.78	9.05	9.46				
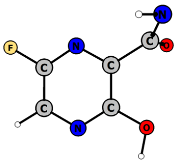	23.27	23.37	22.99				
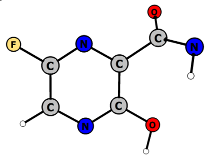	23.50	23.70	23.44				
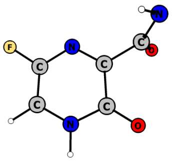	24.50	24.87	24.38				
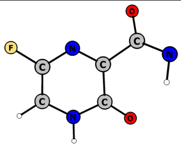	25.06	25.66	25.26				

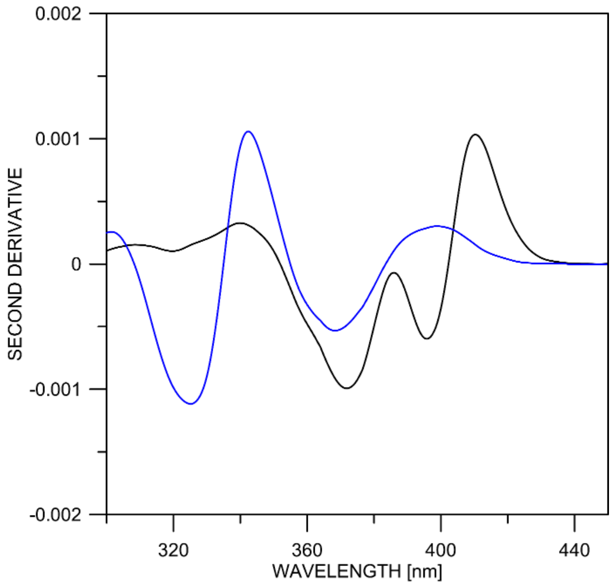

Figure S3. Second derivative spectra of the curves corresponding to the deprotonated **1** in acetonitrile (black line, derived from curve 9 from Figure S2) and **1** with maximum water addition (blue line, derived from curve 5 from Figure 2).

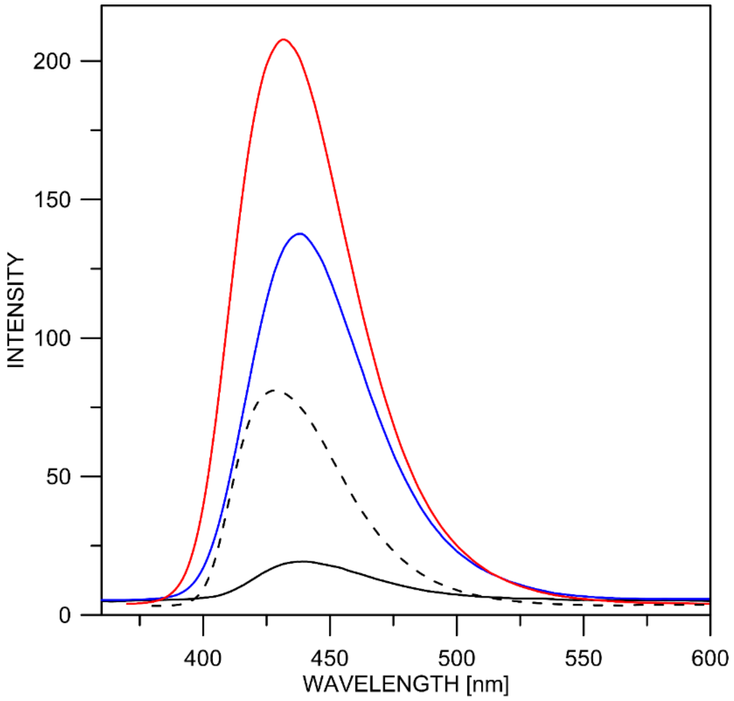

Figure S4. Emission spectra of **1** in acetonitrile (black line) with excitation at 360 nm, in acetonitrile with TEA addition (black dash line) with excitation at 370 nm, in acetonitrile/water mixture: 20%/80% (blue line) with excitation at 320 nm and in acetonitrile/water mixture: 20%/80% (red line) with excitation at 360 nm.

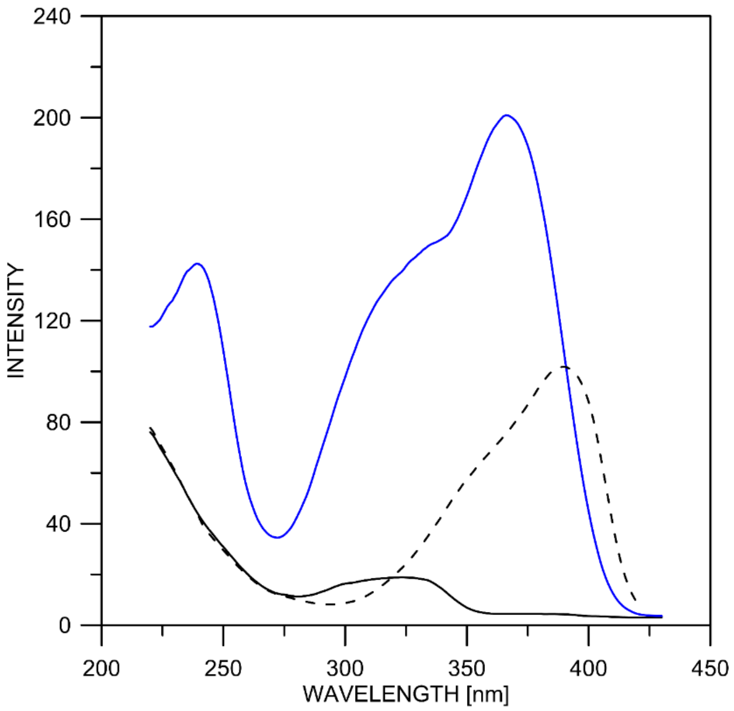

Figure S5. Excitation spectra of **1** in acetonitrile (black line) with emission at 440 nm, in acetonitrile with TEA addition (black dash line) with emission at 430 nm and in acetonitrile/water mixture: 20%/80% (blue line) with emission at 440 nm.

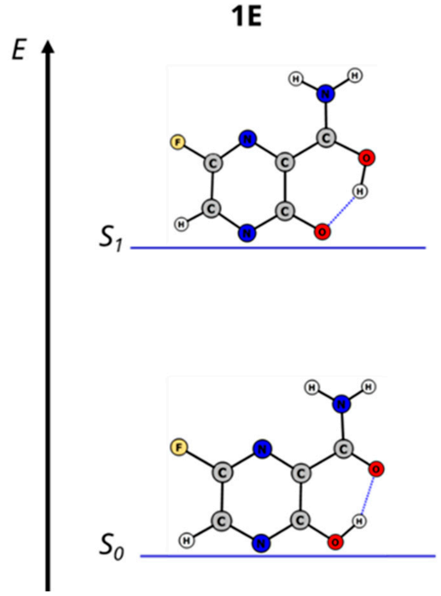

Figure S6. The optimized structures of enol form **1E** of favipiravir in the ground state (S_0, M06-2X/def2-TZVPPD) and its phototautomer in the excited state (S_1, CAM-B3LYP/def2-TZVPPD). The calculations are done in acetonitrile.

Table S4. Emission maxima and quantum yields of favipiravir in different solvents.

solvent	λ^{em}_{max} [nm]	φ
toluene	445	0.003
acetonitrile	438	0.012
chloroform	440	0.006

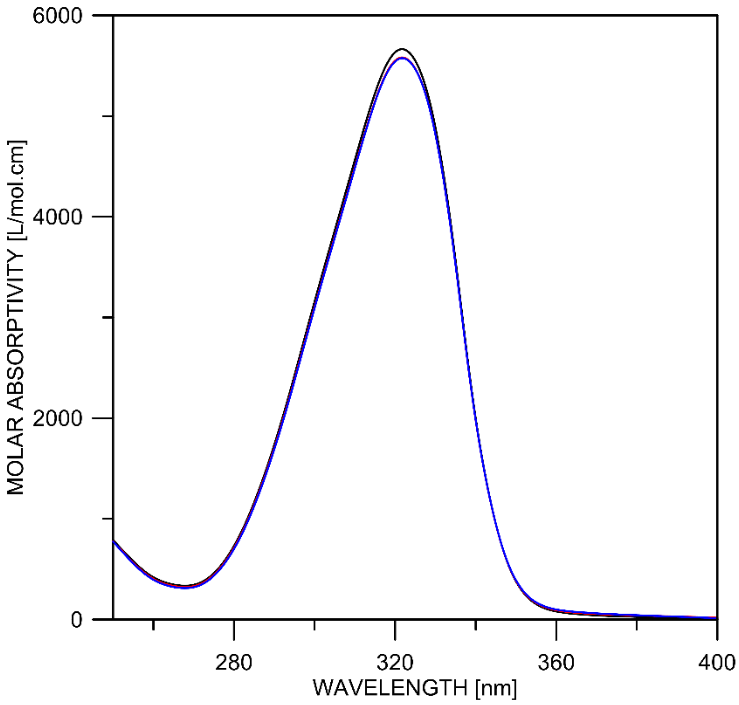

Figure S7. Absorption spectra of favipiravir (c=1.5 x 10^{-5} M) in acetonitrile with $LiClO_4$ addition with molar ratios as follows: 1 (black line) – 100% acetonitrile, 2 (blue line) – 1:2, 3 (red line) – 1:3.

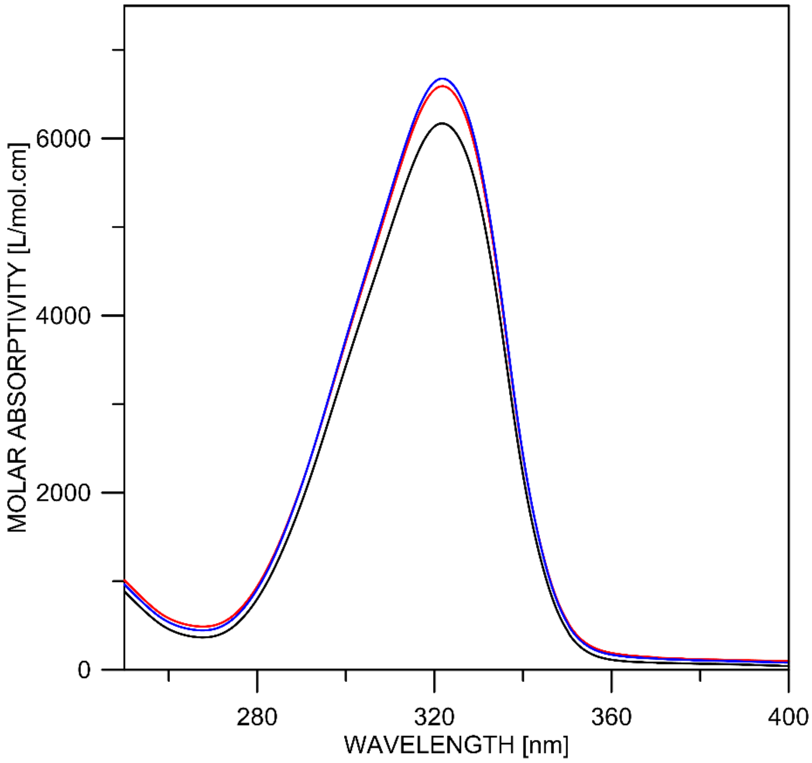

Figure S8. Absorption spectra of favipiravir (c=1.5 x 10^{-5} M) in acetonitrile with $NaClO_4$ addition with molar ratios as follows: 1 (black line) – 100% acetonitrile, 2 (blue line) – 1:2, 3 (red line) – 1:3.

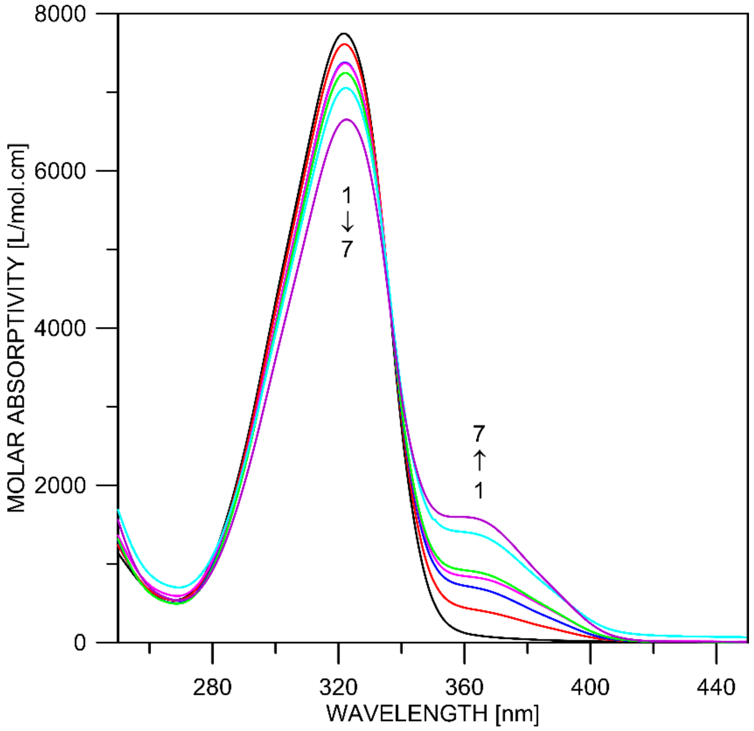

Figure S9. Absorption spectra of favipiravir (c=1.5 x 10^{-5} M) in acetonitrile with $Ca(ClO_4)_2$ addition with molar ratios as follows: 1 (black line) – 100% acetonitrile, 2 (orange line) – 1:0.002, 3 (blue line) – 1:0.02, 4 (red line) – 1:0.1, 5 (green line) – 1:0.2, 6 (cyan line) - 1:0.5, 7 (purple line) – 1:1.

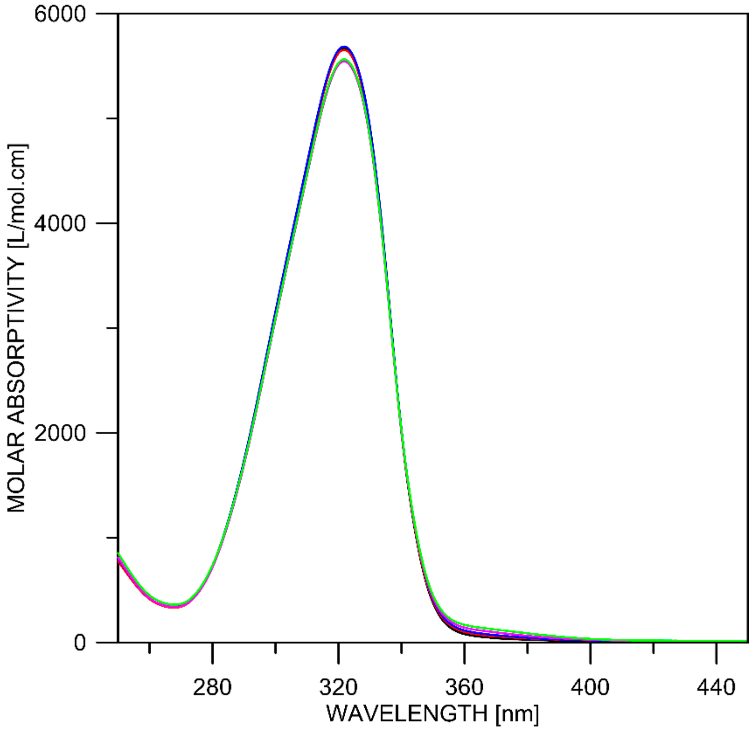

Figure S10. Absorption spectra of favipiravir (c=1.5 x 10^{-5} M) in acetonitrile with $Ba(ClO4)_2$ addition with molar ratios as follows: 1 (black line) – 100% acetonitrile, 2 (red line) – 1:0.002, 3 (blue line) – 1:0.02, 4 (magenta line) – 1:2, 5 (green line) – 1:3.

Table S5: Molar fractions of the Mg^{2+} complex with **1** in acetonitrile (Figure 5).

**Solution**	X_{ligand} [%]	$X_{complex}$ [%]
**2**	95	5
**3**	92	8
**4**	86	14
**5**	77	23
**6**	71	29

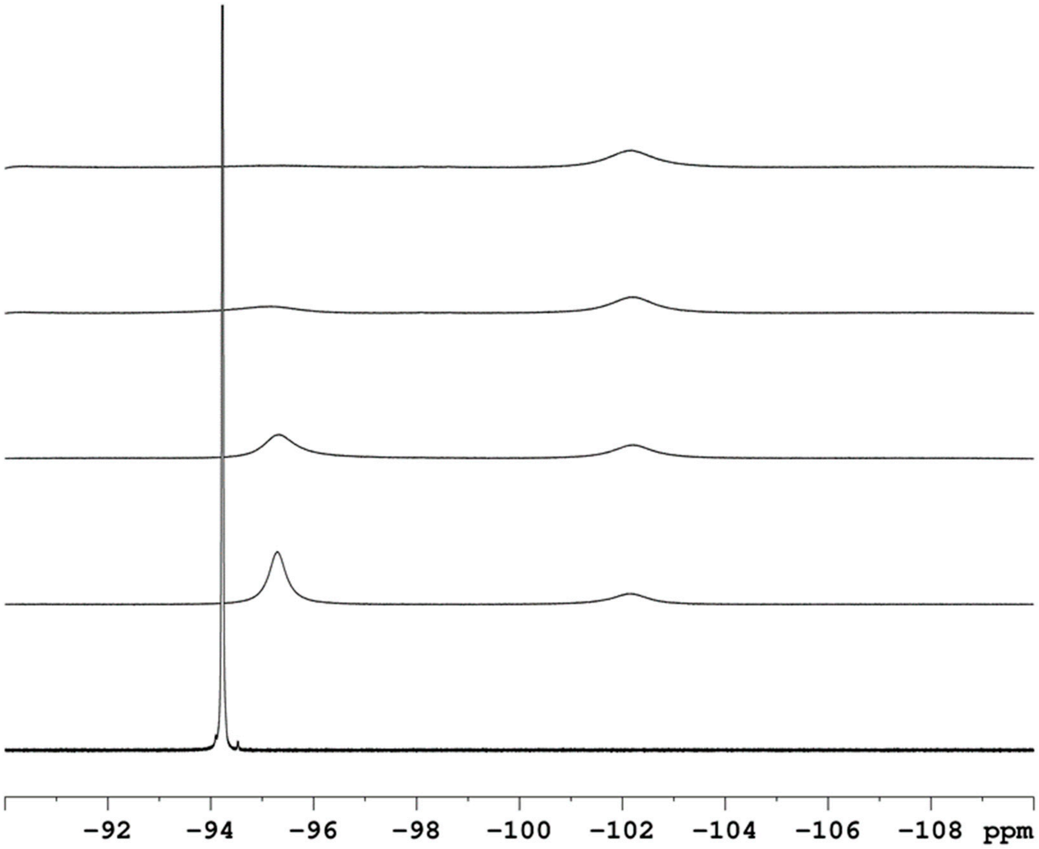

Figure S11. ^{19}F NMR spectra of favipiravir in acetonitrile-d3 with increasing addition of $Mg(ClO_4)_2$ from bottom to top.

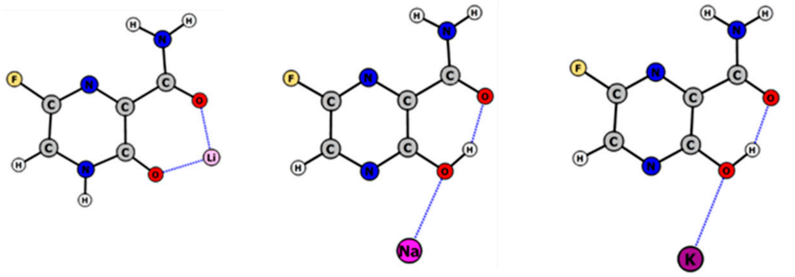

1K′ **1E** **1E**

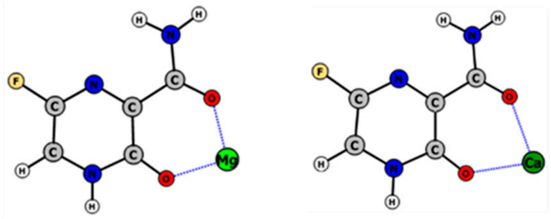

1K′ **1K′**

Figure S12. Theoretically predicted (M06-2X/def2-TZVPPD) most suitable sites for favipiravir complexation with Li^+, Na^+, K^+, Mg^{2+} and Ca^{2+} in acetonitrile in the ground state (S_0).

Table S6. Stabilization energy (M06-2X/def2-TZVPPD, in acetonitrile) of the metal complexes with favipiravir, compared with the ion radiuses of the metal ions (the energy is calculated as $E_{stab}=(E_{lig}+E_{Me})-E_{complex}$)).

Structure	Ion radius [Å]	Ion radius/charge	E_{stab} [kcal/mol]
**1K'-Mg^{2+}**	0.59	0.30	40
**1K'-Ca^{2+}**	0.99	0.50	23
**1K'-Li^{+}**	0.59	0.59	14
**1E-K^{+}**	1.33	1.33	14
**1E-Na^{+}**	0.99	0.99	13
